# Risk of major myopia-associated non-communicable ocular health disorders in Ghana

**DOI:** 10.1371/journal.pone.0297052

**Published:** 2024-01-18

**Authors:** Samuel Kyei, Rexford Kwasi Gyaami, John Baptist Abowine, Ebenezer Zaabaar, Kofi Asiedu, Samuel Bert Boadi-Kusi, Jacob Mensah Mesuh, Frank Assiamah, Anthony Armah, Patience Ansomah Ayerakwah

**Affiliations:** 1 Department of Optometry and Vision Science, College of Health and Allied Sciences, University of Cape Coast, Cape Coast, Ghana; 2 Biomedical and Clinical Research Center, College of Health and Allied Sciences, University of Cape Coast, Cape Coast, Ghana; 3 Dr Rose Mompi Eye Hospital, Hohoe, Volta Region, Ghana; 4 Eye Clinic, Ho Municipal Hospital, Ho, Ghana; Alexandria University Faculty of Medicine, EGYPT

## Abstract

**Objective:**

To assess the differential association of myopia with major non-communicable ocular diseases in an African clinical cohort.

**Methods:**

A five-year hospital-based retrospective study of myopia cases. Patients’ folders, Optical Coherence Tomography scans, and fundus photographs were reviewed for the abstraction of relevant data. Only records that employed recognized standards and classification systems for diagnosing and staging the various ocular conditions were included. Demographic characteristics, non-cycloplegic objective refractive findings, and non-communicable eye diseases were retrieved from the records. Myopia-associated risk factors were then determined using logistic regression and correlation.

**Results:**

Some 16018 patients (32027 eyes) met the inclusion criteria for at least one eye comprising 50.8% males (n = 8137) and 49.2% females (n = 7881). The mean age of the patients was 43.14 ± 17.88 years (range: 2–98 years). The mean spherical equivalent± Standard deviation for myopia was -2.30±3.23 DS (range: -0.50 to -25DS). Binary logistic regression analysis showed that myopic eyes had a higher odd of AC (OR, 0.53; 95% CI, 0.50–0.57), POAG (OR, 6.0; 95% CI, 5.26–6.82), DR (OR, 10.70; 95% CI, 3.91–29.27) and cataracts (OR, 20; 95% CI, 15.32–26.20) but not dry eye (OR, 0.74, 95% CI, 0.68–0.81), macular degeneration and pterygium (OR, 0.36; 95% CI, 0.32–0.40).

**Conclusion:**

Africans with myopia are more at risk of developing allergic conjunctivitis, cataracts, POAG, and DR but not for dry eye, macular degeneration, and pterygium.

## Introduction

Myopia is a common refractive error that has gained notoriety for its global epidemicity, and contribution to visual impairment, especially among children [1,2]. It is estimated that by 2050, 49.8% (4.758 billion) of the world population will be myopic [3] and of this 9.8% will be highly myopic.

The increasing incidence of myopia across the globe is associated with a high risk of other non-communicable ocular diseases with sight-threatening consequences. These include retinal detachment, cataracts, and glaucoma among others [4]. There is a consensus that myopia’s onset and progression are influenced by genetic and environmental factors [5–9].

According to reports, the demographics of myopia appear to be changing in two major ways that are likely to affect the dynamics of myopia-related visual impairment. First, as countries develop and urbanize, the myopia epidemic will undoubtedly impact the ill-prepared healthcare systems of under-resourced settings in dealing adequately with myopia and its associated complications and co-morbidities.

Secondly, the fact that myopia typically develops in children indicates that it is a lifelong disease. In order to develop evidence-based policies and public health interventions for myopia control and treatment, urgent investigations into the dynamics of myopia across various geographic areas and ethnic groups are required. This will allow efficient use of resources, and greatly benefits resource planning and allocation, particularly in resource constraint settings of the world where health care systems are over-burdened.

In the last two decades, much attention has been paid to communicable diseases leading to a marked reduction in blindness due to infectious eye diseases from 20% to 2% [10]. In spite of this landmark achievement, the global effort to address the NCDs has largely missed the major social and economic impacts of non-communicable eye diseases. This has increased the proportion of treatable blindness due to non-communicable eye diseases [11].

It has been reported that people with vision or eye health disorders are at higher risk of other concomitant NCDs than those without [12]. Therefore, a holistic approach must be adopted to understand, plan and mitigate vision impairment attributable to myopia.

Furthermore, the reports of the association of age, sex, and ethnicity with myopia progression validate the need for studies into less studied ethnicities especially Africans [13]. Hence this study was conducted to bring to light the non-communicable ocular health disorders associated with myopia among an African clinical cohort.

## Materials and methods

The study was a five-year hospital-based retrospective study at the Agarwal eye hospital, in Ghana. The facility has a full complement of eye care staff offering a wide range of refractive error services which include; clinical refraction, surgery, spectacle, and contact lens care.

It is one of the leading eye hospitals in Ghana receiving referral cases across the country apart from patients attending appointments or on walk-in visits. Myopia cases were noted in the refractive error cases that were retrieved from patients’ records from January 2016 to December 2020. The data collection was done between July 2022 to December 2022.

### Data collection procedure

For each case of myopia, the autorefractor’s objective refractive findings were recorded for each eye. Myopia was defined as ≥-0.50D <-6.00D and high myopia as -6.00D or worse. Refractive measures were converted into spherical equivalents computed as spherical value plus half the astigmatic value [(sphere +0.5(cylinder)]. Emmetropia was defined as <-0.25D< +0.50D. The objective refraction was non-cycloplegic and the demographic parameters extracted included; age, sex, and other non-communicable eye diseases.

### Diagnostic criteria

POAG included individuals with glaucomatous optic nerve head changes on assessment (diffuse or localized rim thinning and disc hemorrhage, notch, bayoneting, baring or vertical cup-to-disc ratio [˃0.5 or difference in cup disc ratio of more than 0.2 in the two eyes, in the absence of significant difference in disc size) and presence of glaucomatous visual field defects that corresponded with the RNFL defects, optic nerve head abnormalities and gonioscopically open angles.

A non-mydriatic Auto Fundus Camera AFC-330 (NIDEK Co., Ltd, Japan) was used to obtain colour photographs centered at each eye’s optic disc and macula. The retinal images were graded using information from all modalities (fundus photography, OCT, infrared imaging, and autofluorescence). The META-PM grading was performed on the color fundus photographs and the E3 OCT classification was used to grade the OCTs [14,15].

DR was diagnosed based on Fundus photographs graded in a masked manner by two graders according to the modified Airlie House Classification system [16]. The level of retinopathy was graded according to the following criteria: (1) no DR (levels 10–20); (2) NPDR [mild (levels 31–37), moderate (levels 43–47), or severe (levels 53] or (3) proliferative DR (levels 60–85).

Cataracts were diagnosed clinically using the Lens Opacity Classification System (LOCS) III system [17]. The LOCS III included an assessment of nuclear opalescence (NO), cortical cataract (C), and PSC (P). A LOCS III score of ≥4.0 for NO was defined as a significant nuclear cataract, a score of ≥2.0 for C was defined as a significant cortical cataract, and a score of ≥2.0 for P was defined as a significant PSC.

Dry eye was diagnosed based on the following three components: subjective symptoms, tear function (TBUT), and vital staining test [18]. Subjects who were positive for at least two of the above three conditions were regarded as having a dry eye disease.

Allergic conjunctivitis in this study encompasses seasonal allergic conjunctivitis (SAC), perennial allergic conjunctivitis (PAC), vernal keratoconjunctivitis (VKC), and atopic keratoconjunctivitis (AKC). The diagnostic features consisted of itching, redness, swelling of the conjunctiva, and corneal involvement under slit-lamp examination [19], and the diagnosis of pterygium was made clinically under the slit lamp.

### Statistical analysis

The extracted data were screened for missing variables, processed, and analyzed with IBM SPSS version 25 (SPSS Inc., Chicago, IL, USA). The myopia-associated risk factors were determined using logistic regression analysis and correlation analysis and the commodities were expressed as frequencies. P≥0.05 was considered statically significant.

### Ethical consideration

The study protocol was approved by the Institutional Review Board of the University of Cape Coast (UCCIRB/CHAS/2022/40). Permission was sought and obtained from the management of the Dr. Agarwals Eye Hospital and patient anonymity and confidentiality of medical records were ensured by assigning alphanumeric codes to the patients’ records and omitting their dates of birth, addresses, and contact details. This resolved the complex of continuous access to information that could identify patients. Patient information was only used to classify participants into their sexes during the period of data collection. The need for consent was waived since the study involved patients’ records. The study complied with the tenets of the Declaration of Helsinki.

## Results

A total of 16018 patients met the inclusion criteria for at least one eye comprising 50.8% males (n = 8137) and 49.2% females (n = 7881). In all, 32027 eyes of eligible right and left eyes combined were included in the analysis. Overall, the mean age of the patients was 43.14 ± 17.88 years (range: 2–98 years). For myopes, the mean age was 40.45 ± 20.92 years (range: 3–97 years), 49.41 ± 16.40 (range: 2–98 years) for hyperopes, and 39.57 ± 16.32 (range: 3–74 years) for emmetropes. The mean spherical equivalent± Standard deviation for myopia was -2.30±3.23 DS (range: -0.50 to -25DS). The hyperopes had a mean spherical equivalent of +1.40±1.5 DS (range: +0.75 to +20.50DS). Table 1 summarizes the distribution of demographic and ocular co-morbidity variables based on refractive states.

**Table 1:** Associations of refractive states with age, sex, and ocular comorbidities.

		Myopia n (%)	Hyperopia n (%)	Emmetropia n (%)	P
Age		-	-	-	<0.001*
Sex	F	6910 (43.9)	4652 (29.5)	4183 (26.6)	0.243†
	M	7287 (44.75)	4692 (28.82)	4303 (26.43)	
Allergic conjunctivitis	Yes	2800 (43.3)	873 (14.1)	2508 (40.6)	<0.001†
	No	11398	8470	5978	
Dry Eye	Yes	1237 (44.5)	576 (20.7)	964 (34.7)	<0.001†
	No	12961	8767	7522	
Macular degeneration	Yes	92 (83.6)	18 (16.4)	0 (0)	<0.001†
	No	14106	9325	8486	
Diabetic retinopathy	Yes	87 (82.9)	14 (13.3)	4 (3.8)	<0.001†
	No	14111	9329	8482	
POAG	Yes	2482 (73.1)	641 (18.9)	274 (8.1)	<0.001†
	No	11716	8702	8212	
Cataracts	Yes	1831 (58.8)	1228 (39.4)	56 (1.8)	<0.001†
	No	12367	8115	8430	
Pterygia	Yes	680 (21.5)	1459 (46.1)	1028 (32.5)	<0.001†
	No	13518	7884	7458	

*Kruskal-Wallis; †Chi-square.

A binary logistic regression model adjusted for age was fitted to predict the odds for each non-communicable co-morbidity for myopia versus hyperopia using emmetropia as a reference. Allergic conjunctivitis was present in 6181 eyes, of which the mean age of the patients was 31.24 ±17.80 years and ranged from 3–91 years. Compared with emmetropic eyes, myopic eyes had slightly higher odds of allergic conjunctivitis (OR, 0.53; 95%CI, 0.50–0.57) than hyperopic eyes (OR, 0.33; 95% CI, 0.31–0.37) [Table 2].

**Table 2:** Refractive error-related risk factors for major ocular non-communicable co-morbidities adjusted for age.

Co-morbidities	Myopia			Emmetropia			Hyperopia		
	OR	95CI	P	OR	95CI	P	OR	95CI	P
Allergic Conjunctivitis	0.53	0.50–0.57	<0.001	-	Reference	-	0.33	0.31–0.37	<0.001
Dry Eye	0.74	0.68–0.81	<0.001	-	Reference	-	0.51	0.45–0.57	<0.001
Macular Degeneration	5e6	-	0.97	-	Reference	-	1.3e6	-	0.97
Diabetic retinopathy	10.7	3.91–29.27	<0.001	-	Reference	-	2.14	0.70–6.53	0.18
Glaucoma	6.0	5.26–6.82	<0.001	-	Reference	-	1.6	1.35–1.81	<0.001
Cataracts	20.03	15.32–26.20	<0.001	-	Reference	-	15.76	12.02–20.66	<0.001
Pterygia	0.36	0.32–0.40	<0.001	-	Reference	-	1.24	1.14–1.36	<0.001

There were 2777 eyes diagnosed with dry eyes. The mean age of the patients was 42.14 ±16 and range: 5–96 years. Although both myopic and hyperopic eyes were not significant risk factors for dry eye, myopic eyes had higher odds (OR, 0.74, 95% CI, 0.68–0.81) for dry eye than hyperopic eyes (OR, 0.51; 95% CI, 0.45–0.57) in comparison with emmetropic eyes.

Patients diagnosed with aged-related macular degeneration (AMD) had a mean age of 67.83 ± 14.10 years and ranged from 33–90 years. Myopia, just like hyperopia, was not a significant risk factor for AMD.

Diabetic retinopathy (DR) was present in various stages in 105 eyes, and among the patients, the mean age was 58.32 ±17.20 years and ranged 11–97 years. From Table 2, myopic eyes were almost 11 times more likely at risk of DR (OR, 10.70; 95% CI, 3.91–29.27) compared to emmetropic eyes than hyperopic eyes (OR, 2.14; 95% CI, 0.70–6.53).

Moreover, 3397 POAG cases were present Myopic eyes had a higher risk for POAG (OR, 6.0; 95% CI, 5.26–6.82) than hyperopic eyes (OR, 1.56; 95% CI, 1.35–1.81) compared to emmetropic eyes (Table 2).

There were 3115 eyes with cataracts of all forms, and among the patients, the mean age was 53.90 ± 15.97 years and age range of 4–98 years. Myopic eyes had higher odds for developing cataracts (OR, 20; 95% CI, 15.32–26.20) than hyperopic eyes (OR, 15.76; 95% CI, 12.02–20.66).

Pterygium was diagnosed in 3167 eligible eyes of patients with a mean age of 46.46 ± 14.72 and an age range of 15–90 years. In comparison with emmetropia as shown in Table 2, myopic eyes were less likely to develop pterygium (OR, 0.36; 95% CI, 0.32–0.40) unlike hyperopic eyes which had higher risk for pterygium (OR, 1.241; 95% CI, 1.14–1.36).

## Discussion

Risk analysis has proven essential and fundamental in developing interventions for disease conditions. It has been reported risk analysis needs to be done in a manner that demonstrates causal linkage to be useful in laying out preventative measures [20]. In this study, myopes had a higher risk of developing allergic conjunctivitis which is consistent with the study in Taiwan [21]. Animal studies have proven that mast cell degranulation caused by ocular allergic diseases distorts the tight junctions of the cornea. This triggers the exudation of inflammatory cytokines in the cornea consequently leading to retinal inflammation which promotes myopia progression [22]. The main inflammatory cytokines implicated in this mechanistic risk are tumor necrosis alpha (TNF-α) and interleukin-6 (IL-6) apart from the secretions resulting from the degranulation of mast cells. The TNF-α and IL-6 are responsible for the weakening of the tight junctions of the cornea. It is critical to note that the expression levels of TNF-α, IL-6 IL-8, monocyte chemoattractant protein-1, and nuclear factor kappa B are up-regulated in cases of allergic eye diseases but IL-10 and the inhibition of Kappa B are down-regulated [23,24]. There is ample evidence from both epidemiological and animal studies to prove that allergic eye disease influences the pathogenesis of myopia [22].

The risk of dry eye among myopes is unequivocal [25–28]. In this study, myopia was not a risk factor for the development of dry eye despite it having higher odds compared to hyperopia. It can therefore be deduced that dry eye is a non-specific risk factor for myopia [26,29].

A systematic review and meta-analysis indicated a pooled prevalence of myopic macular degeneration as 2.1% (95%, CI:1.3–3.3%) globally. This is an indication that is rare compared to other ocular disorders such as allergic conjunctivitis, and glaucoma among others. The was no observable risk of myopes developing macular degeneration in this study. This could be due to its relatively rare nature. However, a more probable reason could be due to the fact that the mean spherical equivalent for myopia is not high enough to elicit significant macular changes. It has further been shown that the macula complications are related to the severity of myopia such that the complication correlates exponentially per unit decrease in the dioptric sphere of myopes [30].

Although there is overwhelming evidence to indicate that myopia has a protective effect against diabetic retinopathy, epidemiological studies show some inconsistencies [31–35]. Contrary to the huge evidence to support the protective effect of myopia against diabetic retinopathy, myopes in this study were found to be almost 11 times more likely at risk of DR (OR, 10.70; 95% CI, 3.91–29.27) compared to emmetropes and hyperopes (OR, 2.14; 95% CI, 0.70–6.53). This could be due to the relatively low myopic cases in this study. Most studies conducted on the linkage between myopia and DR are population-based unlike the use of the clinical sample in this case. This could possibly be because most of the patients had other systemic co-morbidities. It is recommended that a large-scale population-based study be conducted to clarify this contradiction.

The risk of myopes developing POAG has been well elaborated by several studies [36–39]. This risk increases with an increasing degree of myopia. A number of theories have been espoused as the mechanistic underpinning for the link between myopia-POAG co-morbidity. There exists an increased susceptibility of optic nerve head to damage by elevated intraocular pressure and increased effect of shearing forces in optic nerve damage. Myopic eyes have slightly higher intraocular pressures than their emmetropic and hyperopic counterparts. Optic nerve head damage appears more pronounced in myopes [40,41]. A more recent genetic epidemiological study of adults and aging cohorts performed to ascertain the potential causal effect between myopia and POAG observed that persons with POAG are more likely to have myopia or high myopia. Genetic correlation indicates POAG is correlated with myopia and high myopia [42]. This further supports the need for POAG risk stratification and screening based on refractive status in aggressively tackling the high prevalence of POAG in Ghana.

Myopic people tend to develop cataracts much earlier in life than emmetropes [43]. This study is consistent with other epidemiological studies which indicate myopia as a risk factor for the development of cataracts [44,45]. It has been stated that vitreous syneresis, low glutathione levels, and myopia’s potential damage rod-out segments, down-regulation of CRYAA through hypermethylation of CpG islanders in its promoters could underline the earlier onset of cataracts in high myopes [46]. It has also been postulated that there are localized refractive index changes along cortical spokes opacities with the pupillary area for patients with refractive error who suffer cortical cataracts whiles nuclear cataracts are associated with myopic shift [47].

Myopia as a protective factor for pterygia has been reported over a wide age range [48]. The finding of this study corroborates the outcomes of other studies elsewhere which posit that patients with pterygium were much less likely to be myopic than their age-matched peers [49]. The environmental interplay could be that myopia is associated with indoor activities where exposure to sunlight which has been found to be a major risk factor for the development of pterygia is limited [50,51]. The mechanism for this has been shown to relate to short axial length through SDF-1/CXCRY signaling [52].

In conclusion, Africans with myopia are more at risk of developing allergic conjunctivitis, cataracts, POAG, and DR but not for dry eye, macular degeneration, and pterygium. The findings of this study contribute to understanding the association and potential risk of major non-communicable ocular health disorders in myopes. This will inform the stratification of non-communicable ocular health disorders by myopia thereby enabling a holistic, prompt diagnosis and preventative risk approaches for high-risk individuals.

## Supporting information

S1 File All minimum datasets have been submitted as a supporting information file (All_myo+emm+hyp_DATA.xls). (XLSX)
